# Effect of comprehensive cardiac rehabilitation after heart valve surgery (CopenHeart_VR_): study protocol for a randomised clinical trial

**DOI:** 10.1186/1745-6215-14-104

**Published:** 2013-04-22

**Authors:** Kirstine Laerum Sibilitz, Selina Kikkenborg Berg, Tina Birgitte Hansen, Signe Stelling Risom, Trine Bernholdt Rasmussen, Christian Hassager, Lars Køber, Daniel Steinbrüchel, Christian Gluud, Per Winkel, Lau Caspar Thygesen, Jane Lindschou Hansen, Jean Paul Schmid, Viviane Conraads, Barbara Christina Brocki, Ann-Dorthe Zwisler

**Affiliations:** 1 The Heart Centre, Department of Cardiology, Rigshospitalet, Copenhagen University Hospital, Copenhagen, Denmark; 2 Department of Cardiology, Gentofte Hospital, Gentofte, Denmark; 3 National Institute of Public Health, University of Southern Denmark, Copenhagen, Denmark; 4 Department of Cardiology, Roskilde Hospital, Roskilde, Denmark; 5 The Heart Centre, Heart and Lung Surgical Clinic, Rigshospitalet, Blegdamsvej 9, Copenhagen, 2100, Denmark; 6 Copenhagen Trial Unit, Centre for Clinical Intervention Research, Rigshospitalet, Copenhagen University Hospital, Copenhagen, Denmark; 7 Swiss Cardiovascular Centre Bern, Cardiovascular Prevention and Rehabilitation Unit, University Hospital, Bern, Switzerland; 8 Cardiac Rehabilitation Centre, Heart Failure Clinic and Medical Heart Transplant Follow-up, Department of Cardiology, Antwerp University Hospital, Antwerp, Belgium; 9 Department of Ergo-and physiotherapy, Aalborg University Hospital, Aalborg, Denmark

**Keywords:** Heart valve surgery, Rehabilitation, Physical exercise, Psycho-education

## Abstract

**Background:**

Heart valve diseases are common with an estimated prevalence of 2.5% in the Western world. The number is rising due to an ageing population. Once symptomatic, heart valve diseases are potentially lethal, and heavily influence daily living and quality of life. Surgical treatment, either valve replacement or repair, remains the treatment of choice. However, post surgery, the transition to daily living may become a physical, mental and social challenge. We hypothesise that a comprehensive cardiac rehabilitation programme can improve physical capacity and self-assessed mental health and reduce hospitalisation and healthcare costs after heart valve surgery.

**Methods:**

A randomised clinical trial, CopenHeart_VR,_ aims to investigate whether cardiac rehabilitation in addition to usual care is superior to treatment as usual after heart valve surgery. The trial will randomly allocate 210 patients, 1:1 intervention to control group, using central randomisation, and blinded outcome assessment and statistical analyses. The intervention consists of 12 weeks of physical exercise, and a psycho-educational intervention comprising five consultations. Primary outcome is peak oxygen uptake (VO_2_ peak) measured by cardiopulmonary exercise testing with ventilatory gas analysis. Secondary outcome is self-assessed mental health measured by the standardised questionnaire Short Form 36. Also, long-term healthcare utilisation and mortality as well as biochemistry, echocardiography and cost-benefit will be assessed. A mixed-method design is used to evaluate qualitative and quantitative findings encompassing a survey-based study before the trial and a qualitative pre- and post-intervention study.

**Discussion:**

The study is approved by the local regional Research Ethics Committee (H-1-2011-157), and the Danish Data Protection Agency (j.nr. 2007-58-0015).

**Trial registration:**

ClinicalTrials.gov (http://NCT01558765).

## Background

Heart valve diseases are common with an estimated prevalence of 2.5% in the Western world population [1]. The number is rising due to an ageing population, thus leading to a growing public health problem [2]. Once symptomatic, heart valve diseases affect exercise tolerance. In the case of aortic stenosis, the disease may lead to sudden death. Symptomatic heart valve disease heavily influences the performance of daily living and quality of life. Surgical treatment, either valve replacement or repair, remains the treatment of choice [3]. Following surgery, the transition to daily living may become a physical, mental and social challenge [4-6].

Clinical guidelines emphasise the importance of specialised rehabilitation after valvular surgery [7], although randomised clinical trials on rehabilitation after heart valve surgery are few in number. Current guidelines on cardiac rehabilitation in valvular heart disease are based primarily on results from randomised clinical trials on cardiac rehabilitation in patients with coronary artery disease [8] and congestive heart failure [9]. However, it is questionable whether the results from these trials are transferable to a heart valve surgery population.

Patients undergoing heart valve surgery commonly present with impairment of physical activity and physical capacity up to several years before surgery [10]. Combined with a period of bed rest after surgery, this patient group is therefore not in an optimum state of physical fitness at hospital discharge and physical rehabilitation is required [11].

Previous clinical studies concerning the effect of physical exercise have shown positive impact of exercise training on quality of life and exercise tolerance [12,13] in patients after after heart valve surgery, being safe and not deteriorating the outcome of recent surgery [13]. A small randomised clinical trial by Landry *et al*., including 20 patients after aortic valve replacement, found that exercise capacity measured by work load and peak oxygen uptake (VO_2_ peak) increased by up to 5.0 mL/kg/min (23%) after a physical exercise programme [14]. An observational study by Habel-Verge *et al.* of 19 women after mitral valve replacement, found that mean peak VO_2_ increased by 4.0 mL/kg/min [15]. Newell *et al.* found similar results in a randomised clinical trial with 24 patients after mitral or aortic valve replacement [10]. Functional evaluation studies after heart valve surgery such as conducted by Niemela *et al.* also show improvements in left ventricular ejection fraction and a concomitant decrease in New York Heart Association Class with physical exercise [16].

Furthermore, patients after heart valve surgery can experience difficulties returning to daily living [4,17-19]. Impaired quality of life, depressive symptoms or overt depression, anxiety and post-traumatic stress disorder, can be observed. We therefore hypothesize that consultations focusing on disease management, coping strategies and individually tailored information are needed to better support mental and psychological recovery.

Seven randomised trials have been identified that focused on rehabilitation after heart valve surgery [6,10,14,15,20-22]. However, none of these combine physical exercise training with psycho-educational intervention after heart valve surgery. Furthermore, the trials are narrowly focused, and conducted among highly selected small trial populations with a lower mean age than the average heart valve surgery population. The general applicability of these trials is limited and they must be considered as pilot studies.

Thus, a large-scale randomised trial is needed to investigate the effect of cardiac rehabilitation after heart valve surgery. We therefore designed The CopenHeart_VR_ (VR= valvular replacement or repair) trial to investigate the effect of comprehensive cardiac rehabilitation consisting of both physical exercise and a psycho-educational component. This large-scale randomised clinical trial will ascertain whether comprehensive cardiac rehabilitation is superior to treatment as usual for a broad group of patients undergoing isolated heart valve surgery. Isolated heart valve surgery refers to no simultaneous coronary artery bypass surgery.

## Methods

Major parts of the methods section and trial design in this paper are similar to two other randomised clinical trials, CopenHeart_RFA_ and CopenHeart_IE_, and therefore sections from this paper will be identical in these trial protocols [23,24].

Due to the differences in the three patient groups in the three randomised clinical trials, the intervention and outcome measures differ slightly, most importantly with regard to the psycho-educational intervention, which is longer for patients treated for infective endocarditis, because of the complexity of the disease and the longer hospitalisation. Biochemical markers are similarly chosen differently to address the different co-morbidities of the three different patient groups.

### Ethical considerations

The study is approved by the local regional Research Ethics Committee (H-1-2011-157), and the Danish Data Protection Agency (j.nr. 2007-58-0015), and is registered at ClinicalTrials.gov (http://NCT01558765). Although guidelines recommend cardiac rehabilitation for patients after heart valve surgery, evidence is lacking regarding its effectiveness. The extent of cardiac rehabilitation has been very limited in Denmark, and the local ethics committee agreed with the investigator group that we had a unique opportunity to conduct a high-quality trial with a well-described randomisation and blinded outcome evaluation. All participants supply written informed consent. The trial is conducted in accordance with the latest edition of the Helsinki Declaration, and relevant regulatory requirements.

### Objectives

We hypothesise that comprehensive cardiac rehabilitation after heart valve surgery compared with treatment as usual [7,25,26] improves physical capacity (at 4 months) measured by peak VO_2_ (oxygen uptake) by >3 mL/kg/min (primary outcome) [6,10,14,15,20,21,27], and improves self-rated mental health (at 6 months) measured by Short Form 36 (SF-36), the mental health component scale (secondary outcome) by 7 points [28,29]. The reason for choosing separately assessed outcome times is due to the different durations of the two intervention elements; the exercise training is undertaken until 4 months post surgery and the psycho-educational intervention until 6 months post surgery. Further, the physical recovery is expected to be faster than the mental recovery and therefore not comparable.

In exploratory analyses we will assess the effects of integrated rehabilitation *versus* treatment as usual on a 6-minute walk test, heart specific biomarkers (pro-brain natriuretic peptide and copeptin [30]), and echocardiographic measurements (diastolic function (E/A ratio), left atrial size (mm), left atrial volume (mL)) [31,32]. Furthermore, we will assess the effects on time to return to work after valve surgery, and long-term (24 months) employment resignation [5,6,33], healthcare utilisation and mortality [8], and cost effectiveness [33]. Standardised questionnaires: Hospital Anxiety and Depression Scale [HADS] [34], Quality of life Cardiac Version [QoL-CV], EQ-5D [35], Heart Related Quality of Life [HeartQoL R] [36], International Physical Activity Questionnaire [IPAQ] [37], physical activity score and the Emotion and Health Scale [38] will be applied to ascertain numerous exploratory hypotheses concerning the possible effects of rehabilitation on patient perceived physical and mental health.

### Design

The CopenHeart_VR_ trial is a multidisciplinary randomised clinical superiority trial, allocating participants 1:1 to experimental intervention *versus* treatment as usual, with blinded outcome assessment.

**Figure 1:**
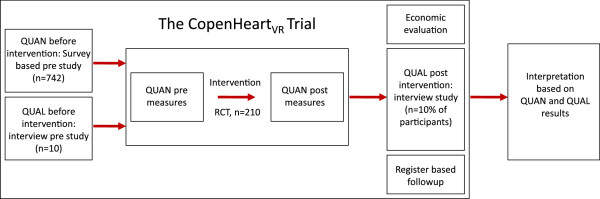
**The CopenHeart**_**VR**_**: trial design.** The CopenHeart_VR_: Mixed methods embedded design comprises qualitative (QUAL) and quantitative (QUAN) data, synthesized in a final analysis.

Alongside the trial we will conduct a number of complementary studies; a survey-based study, to investigate the post-discharge experiences and rehabilitation needs of patients after heart valve surgery (approved by the Danish Data Protection Agency, J.nr. 2011-41-6378); a qualitative interview study of 10 patients aimed at identifying patient experiences and needs after heart valve surgery; a qualitative post-intervention study exploring experiences of participating in a rehabilitation programme; and register-based follow-up after 24 months using appropriate registers described in methods section. Thus, data from the survey based study and the randomised clinical trial are supplemented by qualitative data, and the two methods are integrated by applying a mixed method embedded experimental design (Figure 1). The rationale for this approach is that quantitative findings provide a general understanding of the research problem through statistical results, and subsequent qualitative findings refine and explain the results by exploring participants’ views, thereby facilitating the implementation process after trial completion [39,40].

This paper presents the detailed protocol for the CopenHeart_VR_ trial. The trial is described in accordance with the current SPIRIT guidelines (Standard Protocol Items: Recommendations for Interventional Trials [41]). Results will be reported following the CONSORT (CONsolidated Standards Of Reporting Trials) guidelines for non-pharmacological interventions [42].

### Participants

The trial is planned to include approximately 210 participants of both sexes admitted to hospital for isolated heart valve surgery.

Inclusion criteria are: patients admitted to hospital for elective right-or left-sided heart valve surgery; age ≥18 years; speak and understand Danish; and informed written consent.

Exclusion criteria are: known ischemic heart disease prior to surgery, due to the documented effect of rehabilitation in patients with ischemic disease [8,43]; recruited to other trials including physical exercise and psycho-educational intervention, and participating in other trials inhibiting participating in the present trial; failure to understand and cooperate according to the trial instructions; diseases in the musculoskeletal system or other organs complicating physical activity and exercise training; individuals performing hard physical exercise and competitive sports several times weekly; pregnant and/or breast-feeding women; and lack of informed consent.

### Trial procedure and randomisation

Patient inclusion was initiated on 17 February 2012, and recruitment is currently ongoing. Eligible patients are invited by a nurse or medical doctor to a short consultation on the second or third day after surgery, including distribution of written participant information. Patients needing further time to consider trial participation are contacted by phone 1 week after discharge.

At enrolment, on average 3 to 5 days after surgery, baseline data are collected (Table 1). A 6-minute walk test is performed as close to discharge as possible, using standard guidelines [44]. In case of postoperative complications after enrolment, such as pericardial exudate and atrial fibrillation, the patient’s case will be handled individually, and the intervention will be postponed according to recovery status. Participation in the trial will never delay usual medical follow-up.

**Table 1:** Data collection

	**Baseline**	**1 month**	**4 months**	**6 months**	**12 months**	**24 months**	**Type of data**
*Background*							
Marital status	x						Categorical
Height, weight, body mass index	x						Continuous
*Medical history*							
History of heart disease	x						Binary
Diabetes mellitus	x						Binary
Kidney disease^a^	x						Binary
Chronic obstructive pulmonary disease (COPD)^b^	x						Binary
Hypertension	x						Binary
Dyslipidemia^c^	x						Binary
*Medicine*							
Use of medication, self-reported	x	x	x		x		Categorical
*Heart valve specific questions*							
Type of heart valve disease	x						Categorical
Type of heart valve surgery	x						Categorical
NYHA classification	x	x	x	x	x		Categorical
Left ventricle ejection fraction (LVEF)^d^	x	x	x				Continuous
Euro SCORE II^e^	x						Continuous
Postsurgical complications	x	x					Binary
Postsurgical arrhythmias^f^	x	x	x				Binary
Pacemaker post surgery	x	x	x				Binary
*Clinical measurements*							
Biochemical screening	x	x	x		x		Continuous
ECG		x	x				Binary
Echocardiography		x	x				Continuous
*Physical testing*							
Six minute walk test		x	x		x		Continuous
Cardiopulmonary Exercise Testing		x	x		x		Continuous
Sit and stand test		x	x		x		Continuous
*Questionnaires*							
Level of education	x			x			Categorical
Employment status	x			x			Categorical
Smoking	x			x			Binary
SF-36^g^	x	x	x	x	x	x	Continuous
HADS	x	x	x	x	x	x	Continuous
QoL-CV	x	x	x	X			Continuous
EQ-5D^g^	x			X	x	x	Continuous
HeartQoL	x			X	x	x	Continuous
IPAQ		x	x		x	x	Continuous
Physical activity score	x	x	x	X	x	x	Categorical
Emotion and Health Scale	x			X			Continuous
Rehabilitation received^h^				X			Categorical
*Registry data assessment*							
Mortality, causes of death						X	Categorical
Hospitalisation						X	Continuous
Emergency room visits						X	Continuous
Outpatient clinic visits						X	Continuous
Contact with general practitioner						X	Continuous
Use of medication, register-based						X	Categorical
Employment status						X	Categorical

^a^Kidney disease: patient history of kidney disease.

^b^COPD: patient history of COPD, defined by FEV1 <70%.

^c^Dyslipidemia: total plasma cholesterol >5 mmol/L.

^d^At baseline: LVEF is pre surgery.

^e^European System for Cardiac Operative Risk Evaluation includes patient-related factors, cardiac-related factors and operation-related factors to calculate the predicted operative mortality for patients undergoing cardiac surgery.

^f^Atrial fibrillation, atrial flutter, malign arrhythmias.

^g^EQ-5D™ is a standardised instrument for use as a measure of health outcome from the EuroQoL group. ^h^Rehabilitation received: self-reported (Würgler MW *et al*. *Scand Jour Pub Health* 2012, **40:**126**–**132).

HADS, Hospital and Anxiety Depression Scale; HeartQoL, Heart Disease Health-Related Quality of Life; IPAQ, International Physical Activity Questionnaire; QoL-CV, Quality of Life Cardiac Version; SF-36, Short Form 36.

**Figure 2:**
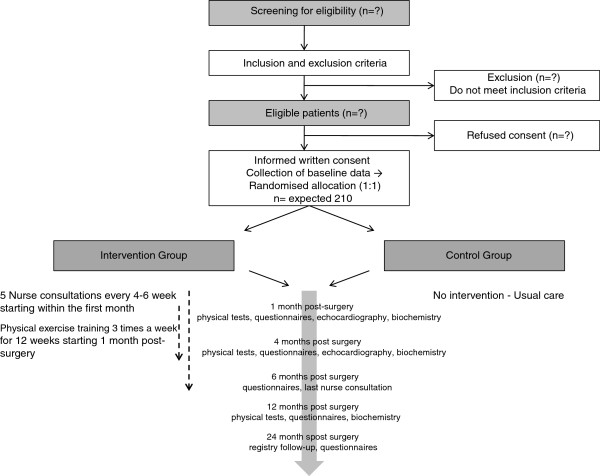
**The CopenHeart**_**VR**_**: trial diagram.** The flow of patients through The CopenHeart_VR_. Participants in the intervention group follow an integrated rehabilitation programme as described, and the control group follow standard care.

After collection of baseline data, central randomisation is conducted by telephoning the Copenhagen Trial Unit. The randomisation is stratified for left ventricular ejection fraction (≥45% or <45%), and type of valve surgery (sternotomy or percutaneous valve surgery, for example, catheter-based surgery), and not for the valve involved. Randomisation is conducted according to a computer-generated allocation sequence with a varying block size, concealed from the investigators to avoid selection bias. Thus, neither investigators and patients nor relatives can influence to which group the patients are allocated. Figure 2 shows patient flow throughout the trial.

Personal information about potential and enrolled patients will be collected electronically and shared in a database accessible only within the project group for those responsible for patient inclusion, in order to protect confidentiality before, during and after the trial.

### Control interventions

Both the intervention and the control group will receive usual care. In accordance with current guidelines a cardiologist sees all patients 4 to 6 weeks after heart valve surgery [45,46]. At the follow-up visit a clinical examination, biochemistry and echocardiography are performed. All patients are given general information on anticoagulation and endocarditis prophylaxis. During the trial period the patients will be seen by a physician at The Heart Centre, Rigshospitalet, for the follow-up visit, after 1 and 4 months. All patients are instructed to initiate their usual activities of daily living. Patients in the control group have accepted not to receive local rehabilitation at the hospital or community setting in their written consent.

### Experimental intervention

The experimental group undergoes the same intervention as described above for the control group. In addition, the experimental intervention consists of a physical exercise training component and a psycho-educational component.

#### The physical exercise component

The CopenHeart intervention has been developed and partly tested in a clinical rehabilitation trial, the COPE-ICD trial [47], including patients with implantable cardioverter defibrillator. We observed a significant impact of the intervention on peak VO_2,_ physical capacity and self-assessed mental health. The intervention has been modified for patients with heart valve surgery as described below.

The CopenHeart physical exercise intervention meets European [48] and Danish guidelines [49] for physical exercise in patients with heart disease, and complies with The National Danish Board of Health recommendations for physical exercise in daily living for heart patients [50].

The physical exercise starts 1 month post surgery after the first cardiopulmonary exercise testing, and comprises the following three elements:

Individual planning of the physical exercise. A specially trained physiotherapist conducts a patient consultation of up to 30 min, integrating detailed information concerning the specific heart valve disease, co-morbidity, hospitalisation, activities of daily living, level of physical activity prior to heart valve surgery, and results from initial testing including cardiopulmonary exercise test 1 month post surgery, a 6-minute walk test, and a ‘sit and stand’ test. The level of physical activity prior to heart valve surgery is also monitored by a self-assessed questionnaire [51]. For all patients, a rehabilitation plan is prepared as an individual training diary based on results from the cardiopulmonary exercise test 1 month post surgery, and the patients are instructed in the use of a heart rate monitor (Polar Watch). The exercise diary and the heart rate monitor recordings are essential in monitoring and in assuring adherence to the intervention. At the end of the intervention the diary and the heart rate monitor are returned and compliance and intensity level are coded independently.

Intensive physical exercise regimen. Physical exercise is initiated at a physiotherapist supervised setting at the Heart Centre, Rigshospitalet, 4 weeks after surgery to ensure optimal healing and decrease the risk of unstable sternum. Using wireless electrodes integrated into t-shirts (Corus-Fit, CardioCardio and Corus Exercise Assistant, version 2.0.16, Finland) potential cardiac arrhythmias, electrocardiographic abnormalities such as ST segment changes, T-wave alterations, atrial or ventricular arrhythmias, and training intensity level are monitored. The training is initiated with one to three mandatory exercise sessions in the primary investigating hospital, Rigshospitalet. Subsequently, the patients can choose to continue the intensive physical exercise regimen either in hospital at Rigshospitalet, at a local CopenHeart-certified facility, supervised by physiotherapists, or as supervised home-based training. Supervised home-based physical training has previously shown similar results to hospital-based training [52]. This finding has been confirmed in a Danish setting [53]. An exercise bicycle at home is required for the patient to perform home-based exercise training.

The training programme continues for 12 weeks, comprising three sessions weekly of 60 min, 36 sessions in total. The training protocol consists of cardiovascular training and strength exercises due to the fact that decreased exercise intolerance in heart valve patients has been suggested to depend on decreased muscular strength [54].

One session consists of 10 min warm-up bicycling, 20 min bicycling with increased intensity (cardiovascular training), 20 min strength exercises, and 10 min stretching and cool-down period. The warm-up session is performed at the intensity of 11–12 on the Borg scale [55]. The 20-min cardiovascular training is performed as interval training. Each session is divided into three sections. Each section contains intensity 13–17 on the Borg scale and time limit (2–15 min) varying between each section; the second section with longest and highest intensity. A cool down period of 5 min is included after the 20 min of cardiovascular training.

The strength and strength-related exercises primarily target lower body muscles, and comprise the following four exercises: (1) Heel rise performed by repetitions of maximal flexion from standing position; (2) Step-up by using a step bench of 27 cm; (3) Leg press standardised, starting with 90 degrees flexion, hyperextension not accepted; (4) 90 degrees pull-down performed in a cable machine to target abdominal muscles. For step-ups and heel-rises, weight load is calculated as a percentage of body weight (5-20%) and increased throughout the 12 weeks. Load for leg press is estimated from repetition maximum (RM) testing and increases from 60% of 1 RM to 70% of 1 RM during the 12 weeks of training. Load for 90 degree pull-downs is decided individually by the physiotherapist assessed from the stabilisation of truncus. All exercises are initiated by 2x12 repetitions and increased through the program according to standard guidelines for strength training [56]. For home-based training two exercises are modified (leg press and abdominal crunch).

Due to the fact of patients having had a sternotomy, upper body strength training is not initiated before the patient is pain free and at least 6 weeks post surgery to avoid complications such as unstable sternum. To achieve cardiovascular adjustment and reduce risk of malignant cardiac arrhythmias, the training sessions are initiated and terminated with a warm-up and cool-down period, with a gradual decrease in training intensity. Training is mainly performed in the upright position to reduce left ventricle preload (diastolic volume).

Sustained moderate physical exercise daily. Patients are guided individually to continue sustained moderate physical exercise daily, and are instructed in maintaining daily moderate physical exercise for at least 30 min during the intervention period and afterwards throughout their lives, for example, bicycling, walking, garden work, jogging or ordinary exercise, and encouraged to use a pedometer [57].

#### The psycho-educational component

The patients receive five consecutive nurse consultations every 4 to 6 weeks during the first 6 months after discharge, initiated within the first month. The consultations take place in a quiet setting at the outpatient clinic or by telephone if the patient is unable to visit the hospital, and are performed by cardiology nurses with specific knowledge of patients with heart valve diseases, after a special training module in the psycho-educational intervention. Using a holistic patient view, the aim of the consultation is to improve patients’ coping strategies, disease management, provide information, and help resume daily life after heart valve surgery. The information given is based on national guidelines and standard treatment of patients after heart valve surgery, and will cover disease management including psychological challenges, and the treatment such as technical and medical questions.

The intervention is inspired by R.R. Parse’s *Human Becoming Practice Methodologies* three dimensions [58] interpreted as: (1) discuss and give meaning to the past, present and future; (2) explore and discuss events and possibilities; and (3) move along with envisioned possibilities. According to the theory, three ways of changing health are possible: (1) creative imaging; that is see, hear and feel what a situation might be like if lived in a different way; (2) affirming personal patterns and value priorities; and (3) shedding light on paradoxes, that is, looking at the incongruence in a situation and changing the view held of something. The nurse is present in the process through discussions, silent immersion and reflection, and is able to facilitate contact to or seek advice from a physician if needed. The method of R.R. Parse was chosen to apply a holistic patient approach centred on the individual person’s themes for the consultations. Furthermore, at The Heart Centre at Rigshospitalet, the method is already extensively used in the outpatient heart clinic, such as for patients with inherited heart diseases and adults with congenital heart disease, and fully documented in the COPE-ICD trial [59]. A consultation guide is used to support the consultation (Table 2). Reported issues for patients after heart valve surgery can be: perceiving fragility, sleeping disturbances, body perception, experiencing an information gap after hospital discharge, and for some, symptoms of depression, anxiety and post-traumatic stress disorder, which will all be covered when relevant [17,18].

**Table 2:** The psycho-educational intervention: consultation guide

**Questions**	**Months after surgery**				
	**1**	**2**	**3**	**4**	**5-6**
Discuss the events leading to heart valve surgery. Experiences before, during and after hospital admission.	x				
Address present thoughts and questions.	x	x	x	x	x
How have you been? What has happened since you were here last time?		x	x	x	x
How did you having heart valve surgery affect your life? Are there things/activities you avoid? Do you in any ways feel impaired after having heart valve surgery?		x		x	x
Have you initiated exercise training? How is training going?		x	x	x	
Discuss social network/family. How do they handle the situation? Has anything changed in your social relationships?	x	x		x	x
Has having heart valve surgery affected your work situation? Has it had financial consequences?				x	
Have you had a changed view/perception of your body and its functions?				x	x
How is your health in relation to fatigue, dyspnea, pain, appetite, gastrointestinal function, sleep, sexual functioning, other?			x	x	x
Symptom handling and degree of dyspnea.	x	x	x	x	x
Information/recommendations in relation to discussed issues/problems according to guidelines or if lacking to usual practice.	x	x	x	x	x

### Intervention deviations

Both the experimental interventions and the control intervention will be supervised regularly to assure protocol compliance. Modification of the allocated intervention due to complications of surgery, rehospitalisation or emerging co-morbidities (for example, pneumonia, pericardial exudation, musculoskeletal problems, atrial fibrillation) is always an individual decision, and the time of the measuring of primary outcome (described in section below) at 4 months will be corrected in accordance with changes in the intervention.

### Outcomes and data collection

#### Primary outcome

Physical capacity is measured after 1 and 4 months by peak VO_2_ using cardiopulmonary exercise testing (Ergo-Spiro CS-200, Schiller, Switzerland), and is performed in accordance with current guidelines [60]. An ergometer bicycle is used, monitoring heart rate, blood pressure, ECG and gas exchange during workload and in the recovery period. Optimal test duration is 8–10 min with a pre- and post-test phase of 2–4 min. Gas, volume and ambient calibration are performed before each session to address changes in room temperature, humidity and air O_2_ content. A ramp protocol is used with initial workload of 25 or 50 watts, increasing by 12.5 watts/min gradually until exhaustion. Exhaustion is evaluated by a respiratory exchange ratio (RER) ≥1.10, reach of anaerobic threshold [60], or the patient’s subjective exhaustion. The peak V0_2_ is determined after standard definitions [60] as the highest V0_2_ measured during the test.

The person who performs the test is either a medical doctor or a nurse with cardiology patient experience. To equally encourage patients independent of the person present, a guide has been developed by the research group, and regular supervision of each tester by a primary investigator is maintained. For safety reasons, criteria for an early test termination have been defined.

#### Secondary outcome

Self-assessed mental health is measured by the mental health component scale of the SF-36 [61] at 1, 4 and 6 months.

#### Exploratory outcome measures

Register-based follow-up. Register data regarding mortality, causes of death, hospitalisation/re-hospitalisation, emergency room visits, outpatient visits, healthcare costs, visits to the general practitioner, medication use, employment status, and payment of welfare benefits (sick leave payment and early retirement pension) will be collected at 24 months to assess the long-term effect of the intervention (Table 1). Danish registers for the data mentioned above function well with only a small percentage of lost data [62]. Consequently the method is well suited as an outcome measure in small patient populations. Data will be extracted from the Danish National Patient Register [63], the Danish National Health Service Register [64], the Danish National Prescription Registry, the Danish National Causes of Death Register [65], and records of transfer payments and labour market affiliation [66,67].

Furthermore, the outcomes below will be measured at 1, 4 and 12 months following randomisation (Table 1). Questionnaires are also distributed at 6 and 24 months.

Six-minute walk test. The maximum walking distance (in meters) within 6 min is measured using standardised instructions [44], while subjective exhaustion with regard to fatigue and dyspnoe before and after using the Borg scale [55] is recorded.

Sit and stand test. The maximum number of times a patient can sit and rise from a normal chair within 30 s. Rate of perceived exertion is measured before and after using the Borg scale.

Biochemical screening. C-reactive protein (CRP), urea, glucose, haemoglobin, HbA1c, potassium, sodium, total cholesterol, creatinine, HDL cholesterol, LDL cholesterol and triglycerides. 1 EDTA plasma heparin tube will be frozen (80°) for further analyses (pro-brain natriuretic peptide and copeptin). The rationale for measuring cholesterol and triglyceride levels is to explore risk markers for cardiovascular disease for this patient group.

Questionnaires. The patient’s perceived physical and mental health will be analysed using relevant standardised questionnaires as mentioned in the objectives section (SF-36, HADS, QoL-CV, EQ-5D, HeartQoL, IPAQ and Emotion and Health Scale).

Echocardiography. Two-dimensional Doppler echocardiography will be performed with standard techniques (Philips iE33/ViCare, Denmark), corresponding to national and international guidelines for a full echocardiography [68] including valve function, with focus on diastolic function (E/A ratio), left atrial size (mm), and left atrial volume (mL). Data will be analysed blinded to randomisation group and time of echocardiography using EchoPac/Excelera.

### Economic evaluation

An economic evaluation will be conducted to assess the cost-utility of cardiac rehabilitation. The economic evaluation will compare the costs to quality-adjusted life years (QALYs), and take a societal perspective as recommended in Danish guidelines. QALYs and costs will be assessed at the end of the intervention 6 months from randomisation and later after 24 months from randomisation using register-based follow-up.

QALYs will be estimated using the self-completed EQ-5D instrument, which is a standardised instrument assessing five dimensions of self-reported health status (mobility, self-care, usual activities, pain/discomfort and anxiety/depression) [69,70]. The estimated calculations will be valued using Danish preference weights [71]. Information on costs will only include costs that are expected to differ between the experimental intervention and treatment as usual group [72]. Costs included costs in the evaluation are health costs associated with the rehabilitation programme, other healthcare costs (healthcare utilisation besides rehabilitation), patient costs, and costs of productivity losses. Information on costs will be collected by a mixture of activity-based costing, surveys, patient diary, and registers.

Results from the analysis will be reported as an incremental cost-effectiveness analysis (ICER). Sensitivity-analyses will be conducted to express uncertainty in the estimates [73]. The reporting of ICER is presented using Bayesian methods, including bootstrapping and presented as cost-effectiveness acceptability curves [74].

### Statistical analysis

SPSS version 17.0 and SAS version 9.3 will be used for the analyses. Data will be pseudo-anonymised and analysed blinded by a trial-independent statistician using intention-to-treat analyses, and a mixed model with repeated measures (MMRM) for continuous outcome measures [75]. Using MMRM ensures that missing data values (in the case of the primary and secondary outcomes) will not create bias as long as the values are missing at random. Two-sided tests are performed. The level of significance is set at 5%. Dealing with multiplicity, gate keeping will be used to adjust the observed *P* values for primary and secondary outcomes [76]. Both unadjusted and adjusted *P* values will be reported.

For the primary and secondary outcome measure, sensitivity analysis will be conducted to assess the potential impact of values missing not at random. For each intervention group (A and B) some quantities (imputing quantities) are computed to be used to impute missing values in a group (A or B) as explained as follows. A comparison between group A and group B where missing values in group A are imputed using imputing quantities obtained from group A and missing values from group B are imputed using imputing quantities obtained from group B is referred to as a best-case analysis. If missing values in group A are imputed using imputing quantities obtained from group B and *vice versa*, the comparison is called a worst-case analysis. The imputing quantities for the primary outcome are the group mean at T1 (X1-bar), the group mean at T4 (X4-bar), the group mean at T6 (X6-bar), the mean difference between the value measured at T4 and that measured at T1 (delta-1), and the mean difference between the value measured at T6 and that measured at T4 (delta-2). The quantities are used to impute missing values in a group (either the same group or the other intervention group) (Table 3). If the standard error (SE) of a parameter estimate calculated using imputed data is smaller than that of the corresponding parameter calculated using complete case data, it is replaced by the latter SE when the *P* value is calculated.

**Table 3:** **The use of imputed values in CopenHeart**_**VR**_

**Observed pattern in group B at 1, 4 and 6 months**	**Imputed value in group B at 1 month**	**Imputed value in group B at 4 month**	**Imputed value in group B at 6 months**
mis^a^, mis, mis	X1-bar^b^	X4-bar^c^	X6-bar^d^
mis, mis, Y3^e^	Y3 - (delta^f^ + delta2^g^)^h^	Y3 - delta2	
mis, Y2, mis	Y2 – delta1		Y2 + delta2
Y1, mis, mis		Y1 + delta1	Y1 + delta1 + delta2
Y1, Y2, mis			Y2 + delta2
Y1, mis, Y3		(Y1 + delta1 + Y3 -delta2)/2	
mis, Y2, Y3	Y2 - delta1		

Table to explain the use of imputing quantities derived from observed values in a group (group A) to impute missing values in a group (group B).

^a^The value at 4 months is missing in group B.

^b^Mean of values observed in group A at 1 month.

^c^Mean of values observed in group A at 4 months.

^d^Mean of values observed in group A at 6 months.

^e^Observed value in group B at 6 months.

^f^The mean of difference between values observed at 4 months and value observed at 1 month in group A.

^g^The mean of difference between value observed at time 6 months and value observed at time 4 months in group A.

^h^If an imputed value is 0 it is set equal to 0.

Mis, Missing value; X1, Value at month 1; X4, Value at month 4; X6, Value at month 6.

Long-term register-based follow-up will be analysed by two different models: non-negative count outcomes (for example, number of contacts to hospital or number of visits to general practitioners) will be analysed by a Poisson model or a zero-inflated Poisson model if the number of zeros are large, and time-to-event data (for example, mortality and leaving the labour market) will be analysed with survival methods (Kaplan-Meier estimator and Cox regression model). Especially for socioeconomic outcomes, competing risks due to mortality will be considered if a large proportion of patients die during follow-up.

Exploratory outcome measures including data from the 6-minute walk test, ‘sit and stand’ test, biochemical screening and echocardiography will be analysed using appropriate statistical methods.

### Sample size and power calculation

We are performing a randomised trial with the continuous variable VO_2_ peak from independent control and intervention group participants as primary outcome with one control per intervention group participant. Previous studies report that VO_2_ peak is normally distributed in the intervention and control groups with a standard deviation between 4.0 and 9.3 mL/kg/min [14,15,20,21], though this might depend on the baseline peak VO2. However, if the true difference between the intervention and control group mean is 3 mL/kg/min [14,15,21], and the standard deviation is 6 mL/kg/min, inclusion of 105 participants is needed in the experimental intervention group, and 105 in the control group (a total of 210 participants) to be able to reject the null hypothesis, stating that the mean in the intervention and the control groups are the same with a power of 95%, and a type I error probability of 5%.

The secondary outcome measure is the continuous variable ‘mental health’. If the true difference between the intervention and control group is 7 points, and the standard deviation is 18 points [28,29], we will be able to reject the null hypothesis that the population means in the intervention and control group are equal with a probability of 80%, and a type 1 error probability of 5%.

## Discussion

The major strength of The CopenHeart_VR_ is that it includes consecutive patients with a reasonable number of inclusion and exclusion criteria securing external validity for the results. The trial employs central, stratified randomisation, which secures against selection bias [77-79]. The primary outcome is assessed blinded to intervention, and so are all statistical analyses, which should reduce detection and interpretation bias [77-79]. The long-term outcomes are based on registry data, which are also likely not to include significantly biased reporting of outcomes.

The secondary outcome of self-assessed mental health is by nature subjective, and is likely to be biased [77-79]. The patients answer questionnaires independently of the researchers. All questionnaires are distributed electronically, thus data management is handled independently from the researchers that interpret data. All data are stored electronically in a coded database, and in an independent spread sheet, only accessible for the CopenHeart Group. The exploratory outcome measures highly impact the importance of this study as very limited knowledge exists concerning social and community outcomes for this patient group.

The limitations of the trial and methods used are similar to those of other trials including physical exercise and physical testing, which are time-of-day, and day-to-day variation in exercise testing [80]. To ensure standard testing of all physical exercise tests in the trial, standardised instructions for patients have been developed as described in the methods section. Including trial patients with left- and right-side heart valve surgery will result in an inhomogeneous trial population. Also, as peak VO2 is dependent on exercise capacity, age or recovery capacities, which differ according to the valve involved, consequently the expected results are going to depend on the recruitment. Conversely, the trial population will be representative of the broad heart valve population, and facilitate the external validity of The CopenHeart_VR_ rehabilitation programme in terms of daily clinical practice.

The fact that patients have different options for their intervention (home-based training, municipal setting or continuous training at a heart centre in hospital setting), and that the effect of the three different settings for training are unknown, might influence the exercise training, and thus the intervention, the primary and secondary outcome and exploratory analyses. However, patient compliance may be increased with different options, and supervised home-based training for cardiac patients has previously shown effective results compared to centre-based training [52,81].

Finally, it has been shown that a substantial number of patients allocated to the control group of a randomised rehabilitation trial perform self-initiated physical exercise training, possibly motivated by the trial information during the recruitment process [82,83].

To conclude, The CopenHeart_VR_ will be unique in obtaining information on the organisation of rehabilitation across sectors, and on how to optimise the transition phase after hospital discharge in order to initiate and provide rehabilitation more rapidly, after heart valve surgery. This is in accordance with current strategies for future healthcare organisation as suggested by the World Health Organization [84]. It is expected that results from the trial will contribute to the development of heart valve specific clinical rehabilitation guidelines, and to identify requirements for resources within the field.

### Safety aspects and Data Monitoring Safety Committee (DMSC)

In supervised exercise training and testing of cardiac patient groups other than post heart valve surgery, the risk of adverse effects is low (for example, ischemic heart disease, chronic heart failure), however, no current national safety instructions exist. Patient safety is given the highest priority, and exercise training after heart valve surgery is considered safe based on results from exercise testing and training with heart failure patients [85,86]. Any serious adverse events will be registered as part of the data collection.

The DMSC works independently from the funder and has no competing interests, and consists of two clinicians and a statistician. The committee is responsible for safeguarding the interests of trial participants, assessing the safety and efficacy of the interventions during the trial, and for monitoring the overall conduct of the clinical trial. The steering committee and the DMSC communicate regularly, and at least every 9 months the overall number of all serious adverse events is reported.

## Trial status

The duration of inclusion is fixed at a maximum of 2 years to minimise the influence of changing treatment trends on usual care intervention over time, and for trial organisational reasons. Based on current activity levels of valvular surgery at the Heart Centre, Rigshospitalet, this can be achieved with an inclusion proportion of 35%. To achieve adequate participant enrolment, patients in doubt are contacted after hospital discharge by phone, and another heart centre that is already in partnership with the trial will be invited to participate if the enrolment rate declines. The inclusion rate is carefully monitored every week.

Results of the trial and complementary studies will be published in relevant international peer-reviewed journals. Authorship will be determined according to the guidelines of the International Committee of Medical Journal Editors. Due to the comprehensiveness of the outcomes the results will be presented in more than one scientific paper as relevant. Economic and long-term follow-up will be reported as data become accessible. Continuously updated information about the trial is available at http://www.copenheart.org/.

## Conclusion

This randomised clinical trial, The CopenHeart_VR_, will be the first trial to investigate the effect of an individually tailored comprehensive cardiac rehabilitation programme comprising physical exercise and a psycho-educational intervention aimed at a broad group of patients after isolated heart valve surgery in a municipal, hospital or home-based setting. Due to its size, positive, negative or neutral outcomes from The CopenHeart_VR_ are likely to have an impact on the organisation of and clinical guidelines for future rehabilitation after heart valve surgery.

## Abbreviations

CONSORT: CONsolidated Standards Of Reporting Trials; COPE-ICD: Copenhagen Outpatient ProgrammE; CopenHeartIE: CopenHeart infective endocarditis; CopenHeartRFA: CopenHeart radiofrequency ablation; CopenHeartVR: CopenHeart valvular replacement or repair; CPET: Cardiopulmonary exercise testing; CRP: C reactive protein; DMSC: Data Monitoring Safety Committee; ECG: Electrocardiogram; EDTA: Ethylenediaminetetraacetic acid; EQ-5D: European Quality of Life 5 dimensions; HADS: Hospital and Anxiety Depression Scale; HbA1c: Glycosylated hemoglobin; HDL: High density lipoprotein; HeartQoL: Heart related Quality of Life; ICER: Incremental cost-effective analysis; IPAQ: International Physical Activity Questionnaire; LDL: Low density lipoprotein; MMRM: Mixed model with repeated measures; RER: Respiratory exchange ratio; RM: Repetition maximum; SAS: Statistical Analysis Software; SE: Standard error; SF-36: Short Form 36; SPIRIT: Standard Protocol Items for Randomized Trials; SPSS: Statistical Package for the Social Sciences; QALY: Quality-adjusted life years; QoL-CV: Quality of life cardiac version

## Competing interests

The authors declare that they have no competing interests.

## Authors’ contributions

ADZ, SKB, KLS, LK, CH, CG, JLH, JPS and VC in collaboration with BCB designed the trial and developed the protocol. KLS in collaboration with ADZ and SKB drafted the manuscript. SSR and TBR contributed significantly during this process. PW and LCT designed and drafted the statistical analysis plan, and TBH designed and drafted the economical analyses plan. KLS, SKB, TBH, TBR, SSR, CH, LK, DS, CG, PW, LCT, JLH, JPS, VC, BCB and ADZ all revised the manuscript critically. All authors have given their final approval of the version to be published.
